# Effect of primary health care on the association between multimorbidity and emergency service utilization: National Health Survey, 2019

**DOI:** 10.1590/1980-549720240062

**Published:** 2024-12-16

**Authors:** Larissa Carolina Xavier Lacerda Lamonato, Thiago Dias Sarti, Ana Paula Santana Coelho Almeida

**Affiliations:** I Universidade Federal do Espírito Santo, Postgraduate Program in Collective Health – Vitória (ES), Brazil.; II Universidade Federal do Espírito Santo, Department of Social Medicine, Center of Health Sciences – Vitória (ES), Brazil.

**Keywords:** Multimorbidity, Primary health care, Health services, Health services accessibility, Health evaluation

## Abstract

**Objective:**

To examine the effect of Primary Health Care (PHC) on the association between multimorbidity and emergency service utilization among adults in Brazil.

**Methods:**

This is a cross-sectional, nationwide household-based study using data from the 2019 National Health Survey. Poisson regression was used to assess emergency service utilization among individuals with multimorbidity. The interaction of variables such as Family Health coverage and orientation to PHC in these associations was also evaluated.

**Results:**

The prevalence of multimorbidity was 31.2% (95%CI 30.9–31.5), Family Health coverage was 71.8% (95%CI 71.4–72.0), and low orientation of services toward PHC was 70% (95%CI 69.1–70.9). Emergency service utilization had a prevalence of 2.0% (95%CI 1.9–2.0), being twice as high among individuals with multimorbidity (3.1; 95%CI 2.9–3.3) compared to those without this condition (1.4; 95%CI 1.3–1.5). However, individuals with multimorbidity and Family Health coverage had a 20% lower prevalence of emergency service utilization than those without Family Health coverage (PR 0.8; 95%CI 0.6–0.9). The association between emergency service utilization and multimorbidity was not modified by the evaluation of the service as highly oriented toward PHC (p=0.956).

**Conclusion:**

The study showed that Family Health coverage exerted a positive effect on the association between multimorbidity and emergency service utilization.

## INTRODUCTION

Chronic noncommunicable diseases (NCDs) are the most prevalent group of diseases in the country, representing a major challenge for primary health care (PHC) services due to their high prevalence and multifactorial origin1. Despite diagnostic and therapeutic advances, control rates for the main NCDs remain low, with these diseases accounting for 54.7% of deaths recorded in Brazil in 2019^1,2^ Accordingly, studies on multimorbidity have gained prominence both in Brazil and internationally. Although there are different definitions in the literature, the most common describes multimorbidity as the presence of two or more diseases in an individual, and the estimated prevalence may vary depending on the definition used^3^. A survey conducted in 14 European countries and Israel revealed a prevalence of multimorbidity of 31.4% (95%CI 30.7–32.2) among people aged 50 or over^4^. In Brazil, the prevalence of multimorbidity in the same age group was estimated at 67.8% (95%CI 65.6–69.9)^5^.

Multimorbidity is considered one of the main predictors of frequent use of emergency services and is strongly associated with increased hospitalizations and emergency room visits. Characteristics such as advanced age, low income, and lower education levels amplify this relationship^6,7^.

As the first level of care and the main gateway to the Brazilian health system, PHC is made up of multidisciplinary teams that cover the entire population, integrating and coordinating care to meet the health needs of people in their area. Playing a strategic role in a polyarchic health care network, PHC is essential for organizing the system and ensuring comprehensive, effective, and accessible care^8,9^.

In Brazil, despite efforts to consolidate PHC and the important results achieved with expanded coverage and effectiveness^10^, there are still obstacles to the operation of a qualified and efficient service that directly impact the health care network^11^. When PHC provides easy access to services, users tend to seek it for less urgent health needs and, by establishing a relationship of trust, are more likely to seek preventive and ongoing care. This scenario, combined with effective care coordination, can mitigate worsening of chronic conditions, acute crises, repeated visits to emergency services, and unnecessary hospitalizations^12–14^.

However, even with a link to PHC, individuals with multimorbidity frequently seek emergency services, suggesting that this condition alone is a significant predictor of the use of these services^6,7^.

Although the literature mentions the influence of PHC characteristics on the use of emergency services, there is still a lack of studies that empirically investigate this relationship or that explore the interaction in greater depth in the context of multimorbidity. Given the above, the present study aimed to determine the effect of PHC on the association between multimorbidity and use of emergency services in adults in Brazil.

## METHODS

A cross-sectional, household-based study was conducted nationwide, using data from the National Health Survey (PNS), conducted by the Ministry of Health and the Brazilian Institute of Geography and Statistics (IBGE) in 2019^15^.

The sample is representative of the Brazilian population living in permanent private households, disaggregated by urban and rural areas, regions, federative units, capitals and metropolitan regions^15^.

A three-stage cluster sampling plan was adopted, in which the census tracts that formed the primary sampling units were initially selected, followed by the selection of households and, finally, of individuals^15,16^.

Data collection was carried out between August 2019 and March 2020 and involved collection agents, supervisors and coordinators. Mobile collection devices were used for the interviews. More details on methodological aspects of the PNS can be found in specific publications^15,16^.

In this analysis, only data from informants aged 18 or over were included. The dependent variable used was the use of emergency services (yes; no), obtained through the following questions: "In the last two weeks, have you sought any place, service or health professional for care related to your own health?" and, if the answer was affirmative, "Where did you seek the first health care for this reason in the last two weeks?". The use of emergency services was considered upon confirmation of care at an emergency care unit or another type of emergency care, emergency room or hospital emergency department, whether public or private.

The main independent variable was multimorbidity (yes; no), defined by two or more affirmative answers from the same individual regarding the occurrence of chronic diseases in the Q module of the questionnaire. Two other important independent variables were Family Health coverage (yes; no), used as a proxy for existence/presence, determined by the affirmative answer to the question: "Is your household registered with the family health unit?"; and the quality of PHC services, analyzed by the degree of orientation toward PHC (high orientation; low orientation), using the General Score obtained through the reduced version of the Primary Care Assessment Tool-Brazil (PCATool-Brazil) for adult patients, available in module H of the questionnaire. High orientation toward PHC was considered in the situation in which the score was ≥6.6, adopting the criterion of the Instrument Application Manual itself, which considers this cutoff point to characterize the presence and extent of PHC attributes^17^.

In the analysis of the association between multimorbidity and emergency service use, Family Health coverage and guidance for PHC were tested as possible effect-modifying variables.

Other independent variables, considered as possible confounding factors, included socioeconomic and demographic data, such as: region (North; Northeast; Southeast; South; Central-West); sex (male; female); skin color or race (White; Black; Brown; Indigenous/Yellow); age group (18–39 years; 40–59 years; 60 years or older); marital status (married; single; separated/divorced; widowed); level of education (no education and incomplete elementary school; complete elementary school and incomplete high school; complete high school and incomplete higher education; complete higher education); occupation (yes; no); per capita household income (no income up to ½ minimum wage; more than ½ to 1 minimum wage; more than 1 to 2 minimum wages; more than 2 to 3 minimum wages; more than 3 minimum wages); health insurance coverage (yes; no); and self-rated health (good/very good; fair; poor/very poor).

The database was obtained from the IBGE website (http://www.ibge.gov.br). Statistical analyses were conducted using Stata software, version 17.0, considering the study's sampling design through the survey module. Data analysis began with descriptive analysis, including the prevalence of the main variables covered in the study with their respective confidence intervals. We also estimated the prevalence of the variables "use of emergency services" and "multimorbidity" according to the independent variables and their respective 95% confidence intervals and p values, with differences assessed by the chi-square test.

For the association analyses, Poisson regression with robust variance was used in seven models, according to the proposed hierarchical model. Initially, the effect of multimorbidity on the outcome was analyzed (Model 1). Family Health coverage was then inserted into the model (Model 2). Model 3 was added to the geopolitical region variable, and Model 4, in turn, had the addition of the variables sex, age group, marital status, education level, occupation and per capita household income. Model 5 included the self-rated health variable. Model 6 was similar to Model 4, with the addition of the health plan coverage variable. Model 7 tested the interaction between multimorbidity and Family Health coverage.

An additional analysis was then performed following the aforementioned hierarchical model, replacing the variable "Family Health coverage" with "orientation toward PHC". In the adjusted models, statistical significance was determined using the Wald test for heterogeneity, with associations with p<0.05 being considered statistically significant.

The PNS was approved by the National Research Ethics Committee under No. 3.529.376.

## RESULTS

A total of 88,531 adults were evaluated. The prevalence of multimorbidity was 31.2% (95%CI 30.9–31.5), Family Health coverage was 71.8% (95%CI 71.4–72.0) and low orientation of services toward PHC was 70% (95%CI 69.1–70.9). The outcome adopted here, which is the use of emergency services, showed a prevalence of 2.0% (95%CI 1.9–2.0) (Table 1).

**Table 1:** Proportional distribution (%) of the sample according to the presence of multimorbidity, Family Health coverage, guidance for primary health care and use of emergency services. National Health Survey, Brazil, 2019.

Variables		%	95%CI
Multimorbidity (n=88,531)			
	Yes	31.2	30.9–31.5
	No	68.8	68.4–69.0
Family Health coverage (n=77,977)			
	Yes	71.8	71.4–72.0
	No	28.2	27.9–28.5
Orientation toward PHC (n=9,479)			
	High orientation	30.0	29.0–30.8
	Low orientation	70.0	69.1–70.9
Use of emergency services (n=88,531)			
	Yes	2.0	1.9–2.0
	No	98.0	97.9–98.1

CI: confidence interval; PHC: primary health care.

The highest prevalence of emergency service use was found in the Southeast region (2.6%; 95%CI 2.3–2.8), by female adults (2.3; 95%CI 2.1–2.4), aged between 40 and 59 years (2.1; 95%CI 1.8–2.2) and 60 years or older (2.2%; 95%CI 2.0–2.3), separated or divorced (2.5; 95%CI 2.1–2.8), covered by health insurance (2.2%; 95%CI 2.0–2.4), with poor or very poor self-rated health (4.4%; 95%CI 3.9–5.0), with multimorbidity (3.1%; 95%CI 2.9–3.3) and without Family Health coverage (2.2%; 95%CI 2.1–2.4) (Table 2).

**Table 2:** Prevalence of use of emergency services and multimorbidity among adults. National Health Survey, Brazil, 2019.

		Use of emergency service		Multimorbidity	
		Prevalence (95%CI)	p-value	Prevalence (95%CI)	p-value
Region (n=88,531)					
	North	1.6 (1.3–1.7)	<0.001	24.4 (23.7–25.0)	<0.001
	Northeast	1.9 (1.7–2.0)		30.6 (30.0–31.0)	
	Southeast	2.6 (2.3–2.8)		36.3 (35.6–36.9)	
	South	1.8 (1.5–2.0)		36.3 (35.4–37.2)	
	Central-West	2.3 (1.9–2.5)		29.4 (28.5–30.2)	
Sex (n=88,531)					
	Male	1.7 (1.5–1.7)	<0.001	23.9 (23.4–24.2)	<0.001
	Female	2.3 (2.1–2.4)		37.8 (37.3–38.2)	
Skin color or race (n=88,522)					
	White	2.0 (1.8–2.1)	0.125	34.8 (34.3–35.3)	<0.001
	Black	2.2 (1.8–2.4)		30.6 (29.6–31.4)	
	Brown	1.9 (1.7–2.0)		28.8 (28.3–29.2)	
	Indigenous/Yellow	2.6 (1.8–3.6)		30.6 (28.1–33.0)	
Age in complete years (n=88,531)					
	18 to 39	1.8 (1.6–1.9)	0.004	12.1 (11.7–12.4)	<0.001
	40 to 59	2.1 (1.8–2.2)		33.7 (33.2–34.2)	
	60 or older	2.2 (2.0–2.3)		55.9 (55.2–56.5)	
Marital status (n=88,531)					
	Married	1.8 (1.7–2.0)	0.001	33.4 (32.8–33.8)	<0.001
	Single	1.9 (1.8–2.0)		21.7 (21.3–22.1)	
	Separated/Divorced	2.5 (2.1–2.8)		41.7 (40.5–42.8)	
	Widowed	2.2 (1.9–2.6)		58.9 (57.8–60.0)	
Level of education (n=88,531)					
	No education and incomplete primary education	1.9 (1.8–2.1)	0.228	40.0 (39.5–40.5)	<0.001
	Complete primary education and incomplete secondary education	2.1 (1.9–2.4)		24.9 (24.0–25.6)	
	Complete secondary education and incomplete higher education	2.0 (1.8–2.2)		23.3 (22.8–23.8)	
	Complete higher education	1.8 (1.6–2.0)		29.8 (29.0–30.6)	
Occupation (n=56,212)					
	Yes	1.9 (1.8–2.0)	0.057	23.0 (22.6–23.3)	<0.001
	No	2.3 (1.9–2.9)		19.1 (17.9–20.4)	
Per capita household income (minimum wage) (n=88,509)					
	No income up to ½	1.8 (1.6–1.9)	0.068	23.6 (23.0–24.1)	<0.001
	More than ½ to 1	2.0 (1.8–2.2)		33.0 (32.4–33.5)	
	More than 1 to 2	2.1 (1.9–2.3)		33.3 (32.7–33.9)	
	More than 2 to 3	2.0 (1.7–2.3)		33.9 (32.7–34.9)	
	More than 3	1.8 (1.6–2.1)		37.0 (36.1–38.0)	
Health insurance coverage (n=88,531)					
	Yes	2.2 (2.0–2.4)	0.016	36.7 (36.0–37.3)	<0.001
	No	1.9 (1.8–2.0)		29.6 (29.2–29.9)	
Self-rated health (n=88,531)					
	Good /very good	1.3 (1.2–1.4)	<0.001	19.0 (18.6–19.2)	<0.001
	Fair	2.6 (2.4–2.8)		46.9 (46.3–47.5)	
	Bad/very bad	4.4 (3.9–5.0)		71.3 (70.1–72.4)	
Family Health coverage (n=77,977)					
	Yes	1.8 (1.7–1.9)	<0.001	32.3 (31.9–32.6)	<0.001
	No	2.2 (2.1–2.4)		30.5 (29.9–31.1)	
Orientation toward PHC (n=9,479)					
	High orientation	1.3 (0.9–1.8)	0.593	55.7 (53.8–57.4)	<0.001
	Low orientation	1.2 (0.9–1.4)		44.9 (43.7–46.1)	

CI: confidence interval; PHC: primary health care.

Higher prevalences of multimorbidity were observed in the Southeast (36.3%; 95%CI 35.6–36.9) and South (36.3%; 95%CI 35.4–37.2) regions, among females (37.8%; 95%CI 37.3–38.2), self-declared white (34.8%; 95%CI 34.3–35.3), aged 60 or over (55.9%; 95%CI 55.2–56.5), widowed (58.9%; 95%CI 57.8–60.0), without education or with incomplete elementary education (40.0%; 95%CI 39.5–40.5), with occupation (23.0%; 95%CI 22.6–23.3), with a per capita household income of more than 3 minimum wages (37.0%; 95%CI 36.1–38.0), covered by health insurance (36.7%; 95%CI 36.0–37.3), with poor or very poor self-rated health (71.3%; 95%CI 70.1–72.4), Family Health coverage (32.3%; 95%CI 31.9–32.6) and who evaluated the service as highly oriented toward PHC (55.7%; 95%CI 53.8–57.4) (Table 2).

Table 3 presents the crude and adjusted models of the association between emergency service use, multimorbidity, and Family Health coverage. In Model 1, it is observed that individuals with multimorbidity were more likely to use emergency services (PR 2.1; 95%CI 1.9–2.3), persisting even after adjustment for Family Health coverage (PR 2.2; 95%CI 1.9–2.3), geopolitical region (PR 2.1; 95%CI 1.9–2.3), and sociodemographic characteristics (PR 2.1; 95%CI 1.8–2.4). In Model 5, when the self-rated health variable was introduced, a significant reduction in the probability of emergency service use was noted (PR 1.7; 95%CI 1.4–1.9). However, when adjusting for health insurance coverage in Model 6, an increase in the prevalence of use was observed (PR 2.1; 95%CI 1.8–2.4), remaining similar to the previous models.

**Table 3:** Crude and adjusted models of the association between multimorbidity and emergency service use among adults. testing the effect of Family Health coverage. National Health Survey. Brazil. 2019.

	PR	95%CI	p-value**
Model 1*	2.1	1.9–2.3	<0.001
Model 2†	2.2	1.9–2.3	<0.001
Model 3‡	2.1	1.9–2.3	<0.001
Model 4§	2.1	1.8–2.4	<0.001
Model 5//	1.7	1.4–1.9	<0.001
Model 6¶	2.1	1.8–2.4	<0.001
Model 7#			0.035

* Model 1: crude;

† Model 2: adjusted for the variable Family Health coverage;

‡ Model 3: adjusted for the variables of Model 2 + geopolitical region;

§ Model 4: adjusted for the variables of Model 3 + sex, age in complete years, marital status, level of education, occupation and per capita household income;

// Model 5: adjusted for the variables of Model 4 + self-rated health;

¶ Model 6: adjusted for the variables of Model 4 + health insurance coverage;

# Model 7: adjusted for the variables of Model 4 + interaction terms;

** Wald test.

Then, it was decided to test, in the adjusted model, the interaction between multimorbidity and Family Health coverage. This showed a significant interaction (p=0.035), suggesting that Family Health coverage changes the use of emergency services by people with multimorbidity.

In Table 4, it is possible to observe that people with multimorbidity and with Family Health coverage had a prevalence of use of emergency services 20% lower than those with multimorbidity and without Family Health coverage (PR 0.8; 95%CI 0.6–0.9).

**Table 4:** Adjusted analysis of the interaction between multimorbidity and Family Health coverage in the association between multimorbidity and emergency service use among adults. National Health Survey, Brazil, 2019.

	PR	95%CI	p-value
Multimorbidity#Coverage*	1		
No#No	0.5	0.3–0.5	<0.001
No#Yes	0.4	0.3–0.4	<0.001
Yes#Yes	0.8	0.6–0.9	0.035

PR: prevalence ratio; CI: confidence interval.

* Model adjusted for the variables geopolitical region, sex, age in complete years, marital status, level of education, occupation, per capita household income.

Table 5, in turn, presents the crude and adjusted models of the association between emergency service use, multimorbidity, and orientation toward PHC among adults. In Model 1, it is observed that individuals with multimorbidity were more likely to use emergency services (PR 2.1; 95%CI 1.9–2.3), even when the variables were adjusted for orientation toward PHC and geopolitical region (PR 2.5; 95%CI 1.7–3.7) or after the inclusion of sociodemographic characteristics (PR 2.2; 95%CI 1.2–4.0).

**Table 5:** Crude and adjusted models of the association between multimorbidity and emergency service use among adults, testing the effect of primary health care orientation. National Health Survey, Brazil, 2019.

	PR	95%CI	p-value#
Model 1*	2.1	1.9-2.3	<0.001
Model 2†	2.5	1.7-3.7	<0.001
Model 3‡	2.5	1.7-3.7	<0.001
Model 4§	2.3	1.3-4.0	0.004
Model 5//	1.6	0.8-2.7	0.116
Model 6¶			0.956

PR: prevalence ratio; CI: confidence interval.

* Model 1: crude;

† Model 2: adjusted for the variable orientation toward primary health care;

‡ Model 3: adjusted for the variables of Model 2 + geopolitical region;

§ Model 4: adjusted for the variables of Model 3 + sex, age in complete years, marital status, level of education, occupation and per capita household income;

// Model 5: adjusted for the variables of Model 4 + self-rated health;

¶ Model 6: adjusted for the variables of Model 4 + interaction terms;

# Wald test.

After the inclusion of the self-rated health variable, in Model 5, the association lost statistical significance. In Model 6, we considered the interaction between multimorbidity and orientation toward PHC. The interaction was not statistically significant (p=0.956), suggesting that the association between emergency service use and multimorbidity is not modified by the assessment of the service as highly PHC-oriented. (Table 5).

## DISCUSSION

The findings of this study revealed that the prevalence of emergency service use was twice as high among individuals with multimorbidity compared to those without this condition. However, lower emergency service use was observed among people with multimorbidity and Family Health coverage compared to those without coverage.

A study reveals a significant increase, at the national level, in the use of emergency care units as a usual source of care, rising from 4.1% in 2013 to 14.1% in 2019^18^. Accordingly, the overload of emergency services has negative consequences for users, staff and the health system as a whole, and there is a consensus among studies that the causes of this problem are mostly outside this level of care^14^.

The high use of emergency services as a regular source of care may indicate a deficiency in access to PHC, which is considered the main gateway to the Brazilian health system, since non-urgent conditions could be treated at other levels of care, and even urgent conditions could be prevented with prior care. However, it is important to recognize that PHC also plays a fundamental role in the care of patients with urgent problems, offering initial diagnostic care, clinical management, stabilization and referral, when necessary. Although not all cases can be prevented, ensuring that patients with non-urgent conditions are treated in PHC can increase the effectiveness of the health care network^19,20^.

The prevalence of emergency service use in this study was higher among women, individuals aged 40 or over, who reported poor or very poor self-rated health, with multimorbidity, health insurance coverage and no Family Health coverage.

A study conducted in Brazil found that women, the elderly and individuals with higher income and education levels had the highest percentages of seeking care and using medical appointments^18^. Regarding the use of emergency services, a study in the South of the country revealed a predominance of use by women, with an average age of 53.37 years, mainly those with chronic diseases^21^. Another investigation conducted in the interior of São Paulo State indicated that the use of these services is more prevalent among women aged 20 to 29, followed by older people aged 60 or over^22^.

Regarding multimorbidity in this study, higher prevalences were observed among women aged 60 or over, with no education or with incomplete elementary education.

The literature indicates that multimorbidity is strongly associated with age, with prevalence increasing significantly from 30% among people aged 45 to 64 years to 65% among those aged 65 to 84 years and reaching 82% among individuals aged 85 years or older. Furthermore, it is more prevalent in women, people with less education and those living in more disadvantaged areas^3^. Low education level was associated with a 64% increased risk of developing multimorbidity^23^.

Higher prevalence of multimorbidity is found among users belonging to lower socioeconomic strata^24^. In the present study, although the prevalence was higher among those with a per capita household income of more than 3 minimum wages, it was observed that this finding was related to the grouping of variables.

Furthermore, a higher prevalence of emergency service use was found among individuals with multimorbidity. The demand for emergency services is influenced by population aging, an increase in the number of individuals with multiple diseases and complex health needs, as well as by socioeconomic factors and political decisions related to the planning and provision of services^13^.

Another study states that older people with multimorbidity use health services more, regardless of sociodemographic factors, highlighting the impact of this condition in determining the use of these services^25^. Thus, older users with complex health conditions and multimorbidity constitute an increasingly important factor in the overload of emergency services^14^.

A higher prevalence of assessment of services as highly oriented toward PHC was also observed among individuals with multimorbidity. This may be related to the fact that the increase in the incidence of diseases implies a greater demand for health services, which, in turn, may result in a better assessment of these services^26^.

In the present study, it was also found that the association between the use of emergency services and multimorbidity was modified by Family Health coverage. Individuals with multimorbidity and Family Health coverage had a lower prevalence of emergency service use than those with multimorbidity but without Family Health coverage.

This result may be related to the increase in PHC coverage over time, favoring access to health services^27^. The expansion of the Family Health Strategy had a significant impact on the health of the Brazilian population, especially in terms of increased access to and use of services, in addition to improved health outcomes, such as reduced infant mortality, malnutrition, and Hospitalizations for Primary Care-Sensitive Conditions (HCSPC)^10,28–30^.

It has also been reported in the literature that the quality of PHC has positive effects, although studies evaluating its association with other health outcomes are scarce.

Studies investigating HCSPC have shown that better results in the quality of PHC resulted in a reduction in these rates, reaffirming the importance of moving beyond expanding coverage. Municipalities with lower quality PHC had 21.2% more HCSPC compared to those with higher quality services^31,32^.

Thus, PHC plays an essential role in promoting health and providing comprehensive care for individuals, especially those with multimorbidity, and can significantly contribute to reducing the use of other levels of care. Strengthening PHC is essential to meet the growing demands of a population with increasingly complex health needs.

However, in this study the association between emergency service use and multimorbidity was not modified by the assessment of the service as highly PHC-oriented. Some factors may be related to this finding.

It is essential to consider that the motivations for using emergency services are complex and multifaceted, and may be influenced by individual and contextual characteristics, as described in the literature^12–14^. In this context, the reduced version of the PCATool-Brazil provides us with information about the services orientation toward PHC, but not necessarily about its ability to meet the individual's health needs. The adoption of a cutoff point of 6.6, despite being established in the literature, may also have corroborated this finding, and may not be sensitive enough to detect subtle differences in certain populations or contexts.

The reduced version of the PCATool-Brazil also has limitations, such as: the absence of analysis of scores by attribute, which makes it difficult to measure the presence and extent of essential and derived attributes of PHC, even through the overall score; and the adaptation of the questionnaire questions, replacing the term "PHC professionals" with the term "physician"^26,33^.

It is important to note that, in the 2019 PNS instrument, all questions related to the PCATool-Brazil refer to the physician who attended the individual in their last appointment at the basic health unit (UBS), and not necessarily to the physician or service to which they are affiliated. This may influence the results obtained in the sense of smoothing out the effect, specifically if the last appointment occurred in another location, where the individual is not affiliated.

Finally, the questions were asked exclusively to people who used the UBS, that is, people covered by the Family Health Service. In this sense, it would be necessary for quality to have a much greater effect to be considered as an effect-modifying variable.

Among the limitations of the study, in addition to those related to the application of the PCATool-Brazil mentioned above, we highlight the use of a cross-sectional design, which prevents the determination of causality between the variables studied. Additionally, the two-week recall period for the use of emergency services may restrict long-term analysis.

Despite the aforementioned limitations, this study stands out for exploring the effect of coverage and orientation of services toward PHC on the relationship between multimorbidity and the use of emergency services, filling an important gap in the literature, using a database representative of the Brazilian population.

The study shows that Family Health coverage is sufficient to modify the effect between multimorbidity and the use of emergency services, in the sense of reducing the use of these services by individuals with multimorbidity. However, in a complex and multifactorial scenario, multimorbidity emerges as a major challenge for health systems, demanding a coordinated effort to address it. Therefore, we suggest that new research be carried out on this topic, which considers the resolution of PHC services themselves, aiming to understand their impact on the health outcomes of individuals with multimorbidity.

## References

[1] Brasil. Ministério da Saúde (2021). Plano de ações estratégicas para o enfrentamento das doenças crônicas e agravos não transmissíveis no Brasil 2021-2030.

[2] Harzheim E, Santos CMJ, D’Avila OP, Wollmann L, Pinto LF (2020). Bases para a reforma da Atenção Primária à Saúde no Brasil em 2019: mudanças estruturantes após 25 anos do Programa de Saúde da Família. Rev Bras Med Fam Comunidade.

[3] Skou ST, Mair FS, Fortin M, Guthrie B, Nunes BP, Miranda JJ (2022). Multimorbidity. Nat Rev Dis Primers.

[4] Nielsen CR, Halling A, Andersen-Ranberg K (2017). Disparities in multimorbidity across Europe – findings from the SHARE survey. Eur Geriatr Med.

[5] Nunes BP, Batista SRR, Andrade FB, Souza-Júnior PRB, Lima-Costa MF, Facchini LA (2018). Multimorbidade em indivíduos com 50 anos ou mais de idade: ELSI-Brasil. Rev Saúde Pública.

[6] Giannouchos TV, Kum HC, Foster MJ, Ohsfeldt RL (2019). Characteristics and predictors of adult frequent emergency department users in the United States: a systematic literature review. J Eval Clin Pract.

[7] Fisher KA, Griffith LE, Gruneir A, Upshur R, Perez R, Favotto L (2021). Effect of socio-demographic and health factors on the association between multimorbidity and acute care service use: population-based survey linked to health administrative data. BMC Health Serv Res.

[8] Brasil. Ministério da Saúde (2017). Departamento de Atenção Básica. Política nacional de atenção básica.

[9] Almeida ER, Sousa ANA, Brandão CC, Carvalho FFB, Tavares G, Silva KC (2018). Política Nacional de Atenção Básica no Brasil: uma análise do processo de revisão (2015–2017). Rev Panam Salud Publica.

[10] Macinko J, Mendonça CS (2018). Estratégia Saúde da Família, um forte modelo de Atenção Primária à Saúde que traz resultados. Saúde Debate.

[11] Melo EA, Mendonça MHM, Oliveira JR, Andrade GCL (2018). Mudanças na Política Nacional de Atenção Básica: entre retrocessos e desafios. Saúde Debate.

[12] Kraaijvanger N, van Leeuwen H, Rijpsma D, Edwards M (2016). Motives for self-referral to the emergency department: a systematic review of the literature. BMC Health Serv Res.

[13] Coster JE, Turner JK, Bradbury D, Cantrell A (2017). Why do people choose emergency and urgent care services? A rapid review utilizing a systematic literature search and narrative synthesis. Acad Emerg Med.

[14] Morley C, Unwin M, Peterson GM, Stankovich J, Kinsman L (2018). Emergency department crowding: A systematic review of causes, consequences and solutions. PLoS One.

[15] Instituto Brasileiro de Geografia e Estatística (2020). Pesquisa Nacional de Saúde 2019: informações sobre domicílios, acesso e utilização dos serviços de saúde: Brasil, grandes regiões e unidades da federação.

[16] Stopa SR, Szwarcwald CL, Oliveira MM, Gouvea ECD, Vieira MLFP, Freitas MPS (2020). Pesquisa Nacional de Saúde 2019: histórico, métodos e perspectivas. Epidemiol Serv Saúde.

[17] Brasil. Ministério da Saúde (2020). Secretaria de Atenção Primária à Saúde. Departamento de Saúde da Família. Manual do instrumento de avaliação da atenção primária à saúde: PCATool – Brasil 2020.

[18] Szwarcwald CL, Stopa SR, Damacena GN, Almeida WS, Souza PRB, Vieira MLFP (2021). Mudanças no padrão de utilização de serviços de saúde no Brasil entre 2013 e 2019. Ciênc Saúde Colet.

[19] Uscher-Pines L, Pines J, Kellermann A, Gillen E, Mehrotra A (2013). Emergency department visits for nonurgent conditions: systematic literature review. Am J Manag Care.

[20] Hong M, Thind A, Zaric GS, Sarma S (2020). The impact of improved access to after-hours primary care on emergency department and primary care utilization: a systematic review. Health Policy.

[21] Acosta AM, Lima MADS (2015). Usuários frequentes de serviço de emergência: fatores associados e motivos de busca por atendimento. Rev Latino-Am Enfermagem.

[22] Garcia VM, Reis RK (2014). Perfil de usuários atendidos em uma unidade não hospitalar de urgência. Rev Bras Enferm.

[23] Pathirana TI, Jackson CA (2018). Socioeconomic status and multimorbidity: a systematic review and meta-analysis. Aust N Z J Public Health.

[24] Andrade FB, Thumé E, Facchini LA, Torres JL, Nunes BP (2022). Education and income-related inequalities in multimorbidity among older Brazilian adults. PLoS One.

[25] Francisco PMSB, Assumpção D, Bacurau AGM, Silva DSM, Malta DC, Borim FSA (2021). Multimorbidade e uso de serviços de saúde em idosos muito idosos no Brasil. Rev Bras Epidemiol.

[26] Carvalho FC, Bernal RTI, Perillo RD, Malta DC (2022). Associação entre avaliação positiva da atenção primária à saúde e características sociodemográficas e comorbidades no Brasil. Rev Bras Epidemiol.

[27] Giovanella L, Bousquat A, Schenkman S, Almeida PF, Sardinha LMV, Vieira MLFP (2021). Cobertura da Estratégia Saúde da Família no Brasil: o que nos mostram as Pesquisas Nacionais de Saúde 2013 e 2019. Ciênc Saúde Colet.

[28] Macinko J, Souza MFM, Guanais FC, Simões CCS (2007). Going to scale with community-based primary care: an analysis of the family health program and infant mortality in Brazil, 1999-2004. Soc Sci Med.

[29] Aquino R, Oliveira NF, Barreto ML (2009). Impact of the family health program on infant mortality in Brazilian municipalities. Am J Public Health.

[30] Monteiro CA, Benicio MHD, Konno SC, Silva ACF, Lima ALL, Conde WL (2009). Causas do declínio da desnutrição infantil no Brasil, 1996-2007. Rev Saúde Pública.

[31] Castro DM, Oliveira VB, Andrade ACS, Cherchiglia ML, Santos AF (2020). Impacto da qualidade da atenção primária à saúde na redução das internações por condições sensíveis. Cad Saúde Pública.

[32] Santos FM, Macieira C, Machado ATGM, Borde EMS, Jorge AO, Gomes BA (2023). Associação entre internações por condições sensíveis e qualidade da atenção primária. Rev Saúde Pública.

[33] Perillo RD, Poças KC, Bernal RTI, Duarte EC, Malta DC (2021). Fatores associados à avaliação da Atenção Primária à Saúde na perspectiva do usuário: resultados do inquérito telefônico Vigitel, 2015. Ciênc Saúde Coletiva.

