# Supplementary material for: Effect of primary health care on the association between multimorbidity and emergency service utilization: National Health Survey, 2019
Source: Rev Bras Epidemiol. 2024 Dec 16;27:e240062. doi: 10.1590/1980-549720240062 (PMC11654288; doi:10.1590/1980-549720240062)
Supplement: Translation

# Translation: Efeito da Atenção Primária à Saúde na associação entre multimorbidade e utilização dos serviços de emergência: Pesquisa Nacional de Saúde (PNS), 2019

**DOI:** 10.1590/1980-549720240062.2

**Authors:** Larissa Carolina Xavier Lacerda Lamonato, Thiago Dias Sarti, Ana Paula Santana Coelho Almeida

**Affiliations:** I Universidade Federal do Espírito Santo, Programa de Pós-Graduação em Saúde Coletiva – Vitória (ES), Brasil.; II Universidade Federal do Espírito Santo, Departamento de Medicina Social, Centro de Ciências de Saúde – Vitória (ES), Brasil.

**Keywords:** Multimorbidade, Atenção primária à saúde, Serviços de saúde, Acesso aos serviços de saúde, Avaliação em saúde

## Abstract

**Objetivo:**

Verificar o efeito da Atenção Primária à Saúde (APS) na associação entre multimorbidade e utilização dos serviços de emergência em adultos do Brasil.

**Métodos:**

Trata-se de um estudo transversal, de base domiciliar em âmbito nacional, sendo utilizados dados da Pesquisa Nacional de Saúde (PNS) de 2019. A regressão de Poisson foi aplicada para avaliar a utilização do serviço de emergência entre pessoas com multimorbidade. Também foi avaliada a interação das variáveis cobertura de Saúde da Família e orientação para a APS nessas associações.

**Resultados:**

A prevalência de multimorbidade foi de 31,2% (IC95% 30,9–31,5), a cobertura de Saúde da Família foi de 71,8% (IC95% 71,4–72,0) e a baixa orientação dos serviços para a APS foi de 70% (IC95% 69,1–70,9). O uso do serviço de emergência apresentou uma prevalência de 2,0% (IC 95% 1,9–2,0), sendo duas vezes maior entre indivíduos com multimorbidade (3,1; IC95% 2,9–3,3) em comparação com aqueles sem essa condição (1,4; IC95% 1,3–1,5). No entanto, pessoas com multimorbidade e cobertura de Saúde da Família apresentaram prevalência de utilização de serviços de emergência 20% menor do que aquelas sem cobertura de Saúde da Família (RP 0,8; IC95% 0,6–0,9). A associação entre o uso do serviço de emergência e a multimorbidade não foi modificada pela avaliação do serviço, como altamente orientado para a APS (p=0,956).

**Conclusão:**

O estudo evidenciou que a cobertura de Saúde da Família exerceu um efeito positivo na associação entre multimorbidade e utilização dos serviços de emergência.

## INTRODUÇÃO

As Doenças Crônicas Não Transmissíveis (DCNT) são o grupo de doenças de maior magnitude no país, representando um grande desafio para os serviços de Atenção Primária à Saúde (APS) devido à sua alta prevalência e à origem multifatorial^1^. Mesmo com o avanço diagnóstico e terapêutico, as taxas de controle das principais DCNT permanecem baixas, com essas doenças correspondendo a 54,7% dos óbitos registrados no Brasil em 2019^1,2^.

Nesse contexto, estudos sobre multimorbidade têm ganhado destaque tanto no Brasil quanto internacionalmente. Embora existam diferentes definições na literatura, a mais comum descreve a multimorbidade como a presença de duas ou mais doenças em um indivíduo, e a prevalência estimada pode variar conforme a definição utilizada^3^. Uma pesquisa realizada em 14 países europeus e em Israel revelou uma prevalência de multimorbidade de 31,4% (IC95% 30,7–32,2) entre pessoas com 50 anos ou mais^4^. No Brasil, a prevalência de multimorbidade na mesma faixa etária foi estimada em 67,8% (IC95% 65,6–69,9)^5^.

A multimorbidade é considerada um dos principais preditores do uso frequente dos serviços de emergência, estando fortemente associada ao aumento das hospitalizações e visitas ao pronto-socorro. Características como idade avançada, baixa renda e menor nível de escolaridade amplificam essa relação^6,7^.

Como primeiro nível de atenção e principal porta de entrada do sistema de saúde brasileiro, a APS é composta por equipes multidisciplinares que cobrem toda a população, integrando e coordenando o cuidado para atender às necessidades de saúde das pessoas em seu território. Desempenhando um papel estratégico em uma rede poliárquica de atenção à saúde, a APS é fundamental para o ordenamento do sistema e para garantir um cuidado integral, resolutivo e acessível^8,9^.

No Brasil, apesar dos esforços para a consolidação da APS e dos importantes resultados alcançados com a ampliação da cobertura e sua efetividade^10^, ainda existem entraves para a operacionalização de um serviço qualificado e eficiente que impactam diretamente a rede de atenção à saúde^11^.

Quando a APS oferece acesso facilitado aos serviços, os usuários tendem a procurá-la para necessidades de saúde menos urgentes e, ao estabelecerem um relacionamento de confiança, são mais propensos a buscar cuidados preventivos e contínuos. Esse cenário, combinado com uma coordenação eficaz do cuidado, pode atenuar agravamentos de condições crônicas, crises agudas, visitas repetidas aos serviços de emergência e internações desnecessárias^12–14^.

No entanto, mesmo com o vínculo com a APS, indivíduos com multimorbidade frequentemente recorrem aos serviços de emergência, sugerindo que essa condição por si só é um preditor significativo para o uso desses serviços^6,7^.

Embora a literatura mencione a influência das características da APS na utilização dos serviços de emergência, ainda há uma escassez de estudos que investiguem empiricamente essa relação ou que explorem mais profundamente a interação no contexto da multimorbidade. Diante do exposto, o presente estudo objetiva verificar o efeito da APS na associação entre multimorbidade e utilização dos serviços de emergência em adultos no Brasil.

## MÉTODOS

Trata-se de um estudo transversal, de base domiciliar em âmbito nacional, sendo utilizados dados da Pesquisa Nacional de Saúde (PNS), realizada pelo Ministério da Saúde e pelo Instituto Brasileiro de Geografia e Estatística (IBGE), em 2019^15^.

A amostra é representativa da população brasileira residente em domicílios particulares permanentes, desagregados por áreas urbanas e rurais, por regiões, Unidades da Federação, capitais e regiões metropolitanas^15^.

Foi adotado plano amostral por conglomerado em três estágios, em que inicialmente foram selecionados os setores censitários que formaram as unidades primárias de amostragem, seguido pela seleção dos domicílios e, por fim, dos indivíduos^15,16^.

A coleta de dados foi realizada entre agosto de 2019 e março de 2020 e envolveu agentes de coleta, supervisores e coordenadores. Para as entrevistas foram utilizados dispositivos móveis de coleta. Mais detalhes sobre aspectos metodológicos da PNS podem ser encontrados em publicações específicas^15,16^.

Nesta análise, foram incluídos somente os dados de informantes com 18 anos ou mais. A variável dependente empregada foi a utilização do serviço de emergência (sim; não), obtida por meio das seguintes perguntas: "Nas duas últimas semanas, procurou algum lugar, serviço ou profissional de saúde para atendimento relacionado à própria saúde?" e, caso respondesse afirmativamente, "Onde procurou o primeiro atendimento de saúde por este motivo nas duas últimas semanas?". A utilização do serviço de emergência foi considerada mediante a confirmação do atendimento em Unidade de Pronto Atendimento ou outro tipo de Pronto Atendimento, Pronto-Socorro ou Emergência de Hospital, seja público ou privado.

A principal variável independente foi a multimorbidade (sim; não), definida mediante duas ou mais respostas afirmativas do mesmo indivíduo sobre a ocorrência de doenças crônicas no módulo Q do questionário. Duas outras importantes variáveis independentes foram a cobertura de Saúde da Família (sim; não), utilizada como *proxy* para a existência/presença, determinada através da resposta afirmativa à pergunta: "O seu domicílio está cadastrado na unidade de saúde da família?"; e a qualidade dos serviços de APS, analisada por meio do grau de orientação para a APS (alta orientação; baixa orientação), utilizando o Escore Geral obtido através da versão reduzida do *Primary Care Assessment Tool*-Brasil (PCATool-Brasil) para pacientes adultos, disponível no módulo H do questionário. A alta orientação para a APS foi considerada na situação em que o escore foi ≥6,6, adotando o critério do próprio Manual de Aplicação do Instrumento que considera esse ponto de corte por caracterizar a presença e a extensão dos atributos da APS^17^.

Na análise de associação entre multimorbidade e utilização do serviço de emergência, a cobertura de Saúde da Família e a orientação para a APS foram testadas como possíveis variáveis modificadoras de efeito.

Outras variáveis independentes, consideradas como possíveis fatores de confusão, incluíram dados socioeconômicos e demográficos, como: região (Norte; Nordeste; Sudeste; Sul; Centro-Oeste); sexo (masculino; feminino); cor ou raça (branca; preta; parda; indígena/amarela); faixa etária (18–39 anos; 40–59 anos; 60 anos ou mais); estado civil (casado; solteiro; separado/divorciado; viúvo); nível de instrução (sem instrução e fundamental incompleto; fundamental completo e médio incompleto; médio completo e superior incompleto; superior completo); ocupação (sim; não); rendimento domiciliar *per capita* (sem rendimento até ½ salário-mínimo; mais de ½ até 1 salário-mínimo; mais de 1 a 2 salários-mínimos; mais de 2 a 3 salários-mínimos; mais de 3 salários-mínimos); cobertura de plano de saúde (sim; não) e autoavaliação de saúde (boa/muito boa; regular; ruim/muito ruim).

A base de dados foi obtida no site do IBGE (http://www.ibge.gov.br). As análises estatísticas foram conduzidas utilizando o *software* Stata, versão 17.0, considerando o desenho amostral do estudo por meio do módulo *survey*. A análise dos dados se iniciou a partir de análise descritiva, incluindo a prevalência das principais variáveis abarcadas no estudo com seus respectivos intervalos de confiança. Também estimamos a prevalência das variáveis "utilização do serviço de emergência" e "multimorbidade" de acordo com as variáveis independentes e seus respectivos intervalos de confiança de 95% e p valores, com as diferenças avaliadas pelo teste qui-quadrado.

Para as análises de associação, foi utilizada a regressão de Poisson com variância robusta empregada em sete modelos, conforme modelo hierárquico proposto. Inicialmente foi analisado o efeito da multimorbidade sobre o desfecho (Modelo 1). Em seguida, a cobertura de Saúde da Família foi inserida no modelo (Modelo 2). O Modelo 3 foi acrescido da variável região geopolítica e o Modelo 4, por sua vez, teve a adição das variáveis sexo, faixa etária, estado civil, nível de instrução, ocupação e rendimento domiciliar *per capita*. No Modelo 5 foi inserida a variável autoavaliação de saúde. O Modelo 6 foi semelhante ao Modelo 4, acrescido da variável cobertura de plano de saúde. No Modelo 7 testou-se a interação entre multimorbidade e cobertura de Saúde da Família.

Em seguida, foi realizada uma análise adicional seguindo o modelo hierárquico supracitado, substituindo a variável "cobertura de Saúde da Família" por "orientação para a APS". Nos modelos ajustados, a significância estatística foi determinada por meio do teste de Wald para heterogeneidade, sendo consideradas estatisticamente significativas as associações com p<0,05.

A PNS teve aprovação da Comissão Nacional de Ética em Pesquisa sob o parecer n° 3.529.376.

## RESULTADOS

Foram avaliados 88.531 adultos. A prevalência de multimorbidade foi de 31,2% (IC95% 30,9–31,5), a cobertura de Saúde da Família foi de 71,8% (IC95% 71,4–72,0) e a baixa orientação dos serviços para a APS foi de 70% (IC95% 69,1–70,9). O desfecho aqui adotado, que é o uso do serviço de emergência, apresentou prevalência de 2,0% (IC95% 1,9–2,0) (Tabela 1).

**Tabela 1:** Distribuição proporcional (%) da amostra segundo presença de multimorbidade, cobertura de Saúde da Família, orientação para a Atenção Primária à Saúde e uso do serviço de emergência. Pesquisa Nacional de Saúde, Brasil, 2019.

Variáveis		%	IC95%
Multimorbidade (n=88.531)			
	Sim	31,2	30,9–31,5
	Não	68,8	68,4–69,0
Cobertura de Saúde da Família (n=77.977)			
	Sim	71,8	71,4–72,0
	Não	28,2	27,9–28,5
Orientação para a APS (n=9.479)			
	Alta orientação	30,0	29,0–30,8
	Baixa orientação	70,0	69,1–70,9
Uso do Serviço de Emergência (n=88.531)			
	Sim	2,0	1,9–2,0
	Não	98,0	97,9–98,1

IC: intervalo de confiança; APS: Atenção Primária à Saúde.

As maiores prevalências de uso do serviço de emergência foram encontradas na região Sudeste (2,6%; IC95% 2,3–2,8), por adultos do sexo feminino (2,3; IC95% 2,1–2,4), com idade entre 40 e 59 anos (2,1; IC95% 1,8–2,2) e 60 anos ou mais (2,2%; IC95% 2,0–2,3), separados ou divorciados (2,5; IC95% 2,1–2,8), cobertos por plano de saúde (2,2%; IC95% 2,0–2,4), com autoavaliação de saúde ruim ou muito ruim (4,4%; IC95% 3,9–5,0), com multimorbidade (3,1%; IC95% 2,9–3,3) e sem cobertura de Saúde da Família (2,2%; IC95% 2,1–2,4) (Tabela 2).

**Tabela 2:** Prevalência de uso do serviço de emergência e multimorbidade entre adultos. Pesquisa Nacional de Saúde, Brasil, 2019.

		Uso do Serviço de Emergência		Multimorbidade	
		Prevalência (IC95%)	p-valor	Prevalência (IC95%)	p-valor
Região (n=88.531)					
	Norte	1,6 (1,3–1,7)	<0,001	24,4 (23,7–25,0)	<0,001
	Nordeste	1,9 (1,7–2,0)		30,6 (30,0–31,0)	
	Sudeste	2,6 (2,3–2,8)		36,3 (35,6–36,9)	
	Sul	1,8 (1,5–2,0)		36,3 (35,4–37,2)	
	Centro-Oeste	2,3 (1,9–2,5)		29,4 (28,5–30,2)	
Sexo (n=88.531)					
	Masculino	1,7 (1,5–1,7)	<0,001	23,9 (23,4–24,2)	<0,001
	Feminino	2,3 (2,1–2,4)		37,8 (37,3–38,2)	
Cor ou raça (n=88.522)					
	Branca	2,0 (1,8–2,1)	0,125	34,8 (34,3–35,3)	<0,001
	Preta	2,2 (1,8–2,4)		30,6 (29,6–31,4)	
	Parda	1,9 (1,7–2,0)		28,8 (28,3–29,2)	
	Indígena/Amarela	2,6 (1,8–3,6)		30,6 (28,1–33,0)	
Idade em anos completos (n=88.531)					
	18 a 39	1,8 (1,6–1,9)	0,004	12,1 (11,7–12,4)	<0,001
	40 a 59	2,1 (1,8–2,2)		33,7 (33,2–34,2)	
	60 ou mais	2,2 (2,0–2,3)		55,9 (55,2–56,5)	
Estado civil (n=88.531)					
	Casado	1,8 (1,7–2,0)	0,001	33,4 (32,8–33,8)	<0,001
	Solteiro	1,9 (1,8–2,0)		21,7 (21,3–22,1)	
	Separado/Divorciado	2,5 (2,1–2,8)		41,7 (40,5–42,8)	
	Viúvo	2,2 (1,9–2,6)		58,9 (57,8–60,0)	
Nível de instrução (n=88.531)					
	Sem instrução e fundamental incompleto	1,9 (1,8–2,1)	0,228	40,0 (39,5–40,5)	<0,001
	Fundamental completo e médio incompleto	2,1 (1,9–2,4)		24,9 (24,0–25,6)	
	Médio completo e superior incompleto	2,0 (1,8–2,2)		23,3 (22,8–23,8)	
	Superior completo	1,8 (1,6–2,0)		29,8 (29,0–30,6)	
Ocupação (n=56.212)					
	Sim	1,9 (1,8–2,0)	0,057	23,0 (22,6–23,3)	<0,001
	Não	2,3 (1,9–2,9)		19,1 (17,9–20,4)	
Rendimento domiciliar *per capita* (salário mínimo) (n=88.509)					
	Sem rendimento até ½	1,8 (1,6–1,9)	0,068	23,6 (23,0–24,1)	<0,001
	Mais de ½ até 1	2,0 (1,8–2,2)		33,0 (32,4–33,5)	
	Mais de 1 a 2	2,1 (1,9–2,3)		33,3 (32,7–33,9)	
	Mais de 2 a 3	2,0 (1,7–2,3)		33,9 (32,7–34,9)	
	Mais de 3	1,8 (1,6–2,1)		37,0 (36,1–38,0)	
Cobertura de plano de saúde (n=88.531)					
	Sim	2,2 (2,0–2,4)	0,016	36,7 (36,0–37,3)	<0,001
	Não	1,9 (1,8–2,0)		29,6 (29,2–29,9)	
Autoavaliação de saúde (n=88.531)					
	Boa/Muito boa	1,3 (1,2–1,4)	<0,001	19,0 (18,6–19,2)	<0,001
	Regular	2,6 (2,4–2,8)		46,9 (46,3–47,5)	
	Ruim/Muito ruim	4,4 (3,9–5,0)		71,3 (70,1–72,4)	
Cobertura de Saúde da Família (n=77.977)					
	Sim	1,8 (1,7–1,9)	<0,001	32,3 (31,9–32,6)	<0,001
	Não	2,2 (2,1–2,4)		30,5 (29,9–31,1)	
Orientação para a APS (n=9.479)					
	Alta orientação	1,3 (0,9–1,8)	0,593	55,7 (53,8–57,4)	<0,001
	Baixa orientação	1,2 (0,9–1,4)		44,9 (43,7–46,1)	

IC: intervalo de confiança; APS: Atenção Primária à Saúde.

Maiores prevalências de multimorbidade foram verificadas nas regiões Sudeste (36,3%; IC95% 35,6–36,9) e Sul (36,3%; IC95% 35,4–37,2), entre pessoas do sexo feminino (37,8%; IC95% 37,3–38,2), autodeclaradas brancas (34,8%; IC95% 34,3–35,3), com 60 anos ou mais (55,9%; IC95% 55,2–56,5), viúvas (58,9%; IC95% 57,8–60,0), sem instrução ou com ensino fundamental incompleto (40,0%; IC95% 39,5–40,5), com ocupação (23,0%; IC95% 22,6–23,3), com um rendimento domiciliar *per capita* de mais de 3 salários-mínimos (37,0%; IC95% 36,1–38,0), cobertas por plano de saúde (36,7%; IC95% 36,0–37,3), com autoavaliação de saúde ruim ou muito ruim (71,3%; IC95% 70,1–72,4), cobertura de Saúde da Família (32,3%; IC95% 31,9–32,6) e que avaliaram o serviço com alta orientação para APS (55,7%; IC95% 53,8–57,4) (Tabela 2).

A Tabela 3 apresenta os modelos bruto e ajustado da associação entre o uso do serviço de emergência, a multimorbidade e a cobertura de Saúde da Família. No Modelo 1, observa-se que os indivíduos com multimorbidade apresentaram maior probabilidade de utilizar o serviço de emergência (RP 2,1; IC95% 1,9–2,3), persistindo mesmo após o ajuste para cobertura de Saúde da Família (RP 2,2; IC95% 1,9–2,3), região geopolítica (RP 2,1; IC95% 1,9–2,3) e características sociodemográficas (RP 2,1; IC95% 1,8–2,4).

**Tabela 3:** Modelos brutos e ajustados da associação entre multimorbidade e uso do serviço de emergência entre adultos, testando o efeito da cobertura de Saúde da Família. Pesquisa Nacional de Saúde, Brasil, 2019.

	RP	IC95%	p-valor**
Modelo 1*	2,1	1,9–2,3	<0,001
Modelo 2†	2,2	1,9–2,3	<0,001
Modelo 3‡	2,1	1,9–2,3	<0,001
Modelo 4§	2,1	1,8–2,4	<0,001
Modelo 5//	1,7	1,4–1,9	<0,001
Modelo 6¶	2,1	1,8–2,4	<0,001
Modelo 7#			0,035

* Modelo 1: bruto;

† Modelo 2: ajustado para a variável cobertura de Saúde da Família;

‡ Modelo 3: ajustado para as variáveis do Modelo 2 + região geopolítica;

§ Modelo 4: ajustado para as variáveis do Modelo 3 + sexo, idade em anos completos, estado civil, nível de instrução, ocupação e rendimento domiciliar per capita;

// Modelo 5: ajustado para as variáveis do Modelo 4 + autoavaliação de saúde;

¶ Modelo 6: ajustado para as variáveis do Modelo 4 + cobertura de plano de saúde;

# Modelo 7: ajustado para as variáveis do Modelo 4 + termos de interação;

** Teste de Wald.

No Modelo 5, ao introduzir a variável autoavaliação de saúde, nota-se uma redução importante na probabilidade de utilização do serviço de emergência (RP 1,7; IC95% 1,4–1,9). No entanto, ao realizar o ajuste para cobertura de plano de saúde, no Modelo 6, evidencia-se um aumento na prevalência de utilização (RP 2,1; IC95% 1,8–2,4), mantendo-se semelhante aos modelos anteriores.

Em seguida, optou-se por testar, no modelo ajustado, a interação entre a multimorbidade e a cobertura de Saúde da Família. Esta apresentou interação significativa (p=0,035), sugerindo que a cobertura de Saúde da Família modifica o uso do serviço de emergência por pessoas com multimorbidade.

Na Tabela 4, é possível observar que pessoas com multimorbidade e com cobertura de Saúde da Família apresentaram prevalência de utilização de serviços de emergência 20% menor que aquelas com multimorbidade e sem cobertura de Saúde da Família (RP 0,8; IC95% 0,6–0,9).

**Tabela 4:** Análise ajustada da interação entre multimorbidade e cobertura de Saúde da Família na associação entre multimorbidade e uso do serviço de emergência entre adultos. Pesquisa Nacional de Saúde, Brasil, 2019.

	RP	IC95%	p-valor
Multimorbidade#Cobertura*	1		
Não#Não	0,5	0,3–0,5	<0,001
Não#Sim	0,4	0,3–0,4	<0,001
Sim#Sim	0,8	0,6–0,9	0,035

RP: razão de prevalência; IC: intervalo de confiança.

* Modelo ajustado para as variáveis região geopolítica, sexo, idade em anos completos, estado civil, nível de instrução, ocupação, rendimento domiciliar *per capita.*

A Tabela 5, por sua vez, apresenta os modelos bruto e ajustados da associação entre o uso do serviço de emergência, a multimorbidade e a orientação para APS entre adultos. No Modelo 1, observa-se que os indivíduos com multimorbidade apresentaram maior probabilidade de utilizar o serviço de emergência (RP 2,1; IC95% 1,9–2,3), mesmo quando as variáveis foram ajustadas para orientação para APS e região geopolítica (RP 2,5; IC95% 1,7–3,7) ou após a inclusão das características sociodemográficas (RP 2,2; IC95% 1,2–4,0).

**Tabela 5:** Modelos brutos e ajustados da associação entre multimorbidade e uso do serviço de emergência entre adultos, testando o efeito da orientação para a Atenção Primária à Saúde. Pesquisa Nacional de Saúde, Brasil, 2019.

	RP	IC95%	p-valor#
Modelo 1*	2,1	1,9-2,3	<0,001
Modelo 2†	2,5	1,7-3,7	<0,001
Modelo 3‡	2,5	1,7-3,7	<0,001
Modelo 4§	2,3	1,3-4,0	0,004
Modelo 5//	1,6	0,8-2,7	0,116
Modelo 6¶			0,956

RP: razão de prevalência; IC: intervalo de confiança.

* Modelo 1: bruto;

† Modelo 2: ajustado para a variável orientação para a Atenção Primária à Saúde;

‡ Modelo 3: ajustado para as variáveis do Modelo 2 + região geopolítica;

§ Modelo 4: ajustado para as variáveis do Modelo 3 + sexo, idade em anos completos, estado civil, nível de instrução, ocupação e rendimento domiciliar *per capita*;

// Modelo 5: ajustado para as variáveis do Modelo 4 + autoavaliação de saúde;

¶ Modelo 6: ajustado para as variáveis do Modelo 4 + termos de interação;

# Teste de Wald.

Após a inclusão da variável autoavaliação de saúde, no Modelo 5, a associação perdeu a significância estatística. No Modelo 6 consideramos a interação entre a multimorbidade e a orientação para a APS. A interação não foi estatisticamente significativa (p=0,956), sugerindo que a associação entre o uso do serviço de emergência e a multimorbidade não é modificada pela avaliação do serviço como altamente orientado para a APS (Tabela 5).

## DISCUSSÃO

Os achados deste estudo revelaram que a prevalência de uso de serviços de emergência foi duas vezes maior entre indivíduos com multimorbidade em comparação com aqueles sem essa condição. No entanto, observou-se menor utilização do serviço de emergência entre as pessoas com multimorbidade e cobertura de Saúde da Família em relação àquelas sem cobertura.

Um estudo revela um aumento significativo, em nível nacional, do uso das Unidades de Pronto Atendimento (UPA) como fonte usual de cuidado, passando de 4,1% em 2013 para 14,1% em 2019^18^. Nesse contexto, a sobrecarga dos serviços de emergência acarreta consequências negativas para os usuários, para a equipe e para o sistema de saúde como um todo e há um consenso entre pesquisas de que as causas para esse problema estão, majoritariamente, fora deste nível de atenção^14^.

A elevada utilização dos serviços de emergência como fonte habitual de cuidado pode indicar uma deficiência no acesso à APS, que é considerada a principal porta de entrada do sistema de saúde brasileiro, uma vez que condições não urgentes poderiam ser tratadas em outros níveis de atenção, e mesmo aquelas de caráter urgente poderiam ser prevenidas com cuidados prévios. Entretanto, é importante reconhecer que a APS também desempenha um papel fundamental no atendimento de pacientes com problemas urgentes, ofertando cuidados iniciais de diagnóstico, manejo clínico, estabilização e encaminhamento, quando necessário. Embora nem todos os casos possam ser evitados, garantir que pacientes com condições não urgentes sejam atendidos na APS pode aumentar a eficiência da rede de atenção à saúde^19,20^.

A prevalência de uso do serviço de emergência neste estudo foi maior entre mulheres, indivíduos com 40 anos ou mais, que referem uma autoavaliação de saúde ruim ou muito ruim, com multimorbidade, cobertura de plano de saúde e sem cobertura de Saúde da Família.

Um estudo realizado no Brasil identificou que mulheres, idosos e indivíduos com maior renda e nível de escolaridade apresentaram os percentuais mais elevados de procura por atendimento e uso de consultas médicas^18^. Em relação à utilização dos serviços de emergência, uma pesquisa no Sul do país revelou uma predominância de uso por mulheres, com média de idade de 53,37 anos, principalmente portadoras de doenças crônicas^21^. Outro estudo realizado no interior de São Paulo indicou que a utilização desses serviços é mais prevalente entre mulheres de 20 a 29 anos, seguidas por idosos com 60 anos ou mais^22^.

Em relação à multimorbidade neste estudo, foram observadas maiores prevalências entre mulheres com 60 anos ou mais, sem instrução ou com ensino fundamental incompleto.

A literatura indica que a multimorbidade está fortemente associada à idade, com a prevalência aumentando significativamente de 30% entre pessoas de 45 a 64 anos para 65% entre aquelas com 65 e 84 anos e alcançando 82% entre indivíduos com 85 anos ou mais. Além disso, é mais prevalente em mulheres, pessoas com menor escolaridade e que vivem em áreas mais desfavorecidas^3^. O baixo nível de escolaridade foi associado a um aumento de 64% no risco de desenvolver multimorbidade^23^.

Maiores prevalências de multimorbidade estão entre usuários pertencentes aos estratos socioeconômicos mais baixos^24^. No presente estudo, apesar de a prevalência ter sido maior entre aqueles com rendimento domiciliar *per capita* de mais de 3 salários-mínimos, observa-se que esse achado está relacionado ao agrupamento das variáveis.

Ainda, constatou-se, entre os indivíduos com multimorbidade, maior prevalência de utilização do serviço de emergência. A demanda pelos serviços de emergência é influenciada pelo envelhecimento populacional, aumento no número de indivíduos com múltiplas doenças e necessidades de saúde complexas, assim como por fatores socioeconômicos e decisões políticas relacionadas ao planejamento e à prestação de serviços^13^.

Outro estudo afirma que idosos com multimorbidade utilizam mais os serviços de saúde, independente de fatores sociodemográficos, destacando o impacto dessa condição na determinação da utilização desses serviços^25^. Dessa forma, usuários idosos, com condições complexas de saúde e multimorbidade constituem um fator cada vez mais importante para sobrecarga dos serviços de emergência^14^.

Também foi observada maior prevalência de avaliação dos serviços como altamente orientados para a APS entre os indivíduos com multimorbidade. Isso pode estar relacionado ao fato de o aumento na incidência de doenças implicar uma maior procura por serviços de saúde, o que, por sua vez, pode resultar em melhor avaliação desses serviços^26^.

No presente estudo foi constatado, ainda, que a associação entre o uso do serviço de emergência e a multimorbidade foi modificada pela cobertura de Saúde da Família. Indivíduos com multimorbidade e cobertura de Saúde da Família apresentaram uma prevalência de utilização de serviços de emergência menor que aqueles com multimorbidade, mas sem cobertura de Saúde da Família.

Esse resultado pode estar relacionado ao avanço da cobertura de APS ao longo do tempo, favorecendo o acesso aos serviços de saúde^27^. A expansão da Estratégia Saúde da Família teve um impacto significativo na saúde da população brasileira, especialmente no que se refere à ampliação do acesso e à utilização dos serviços, além de melhores desfechos de saúde, tais como a redução da mortalidade infantil, desnutrição e Internações por Condições Sensíveis à Atenção Primária (ICSAP)^10,28–30^.

A qualidade da APS também tem referido efeitos positivos na literatura, embora sejam escassos os estudos que avaliam a associação desta com outros desfechos em saúde.

Estudos que investigaram as ICSAP evidenciaram que melhores resultados na qualidade da APS resultaram em redução dessas taxas, reafirmando a importância de se avançar para além da ampliação de cobertura. Municípios com menor qualidade de APS apresentaram 21,2% mais ICSAP em comparação com aqueles com serviços de maior qualidade^31,32^.

Assim, a APS desempenha papel fundamental na promoção da saúde e no cuidado integral dos indivíduos, especialmente daqueles com multimorbidade, podendo contribuir significativamente para a redução da utilização de outros níveis de atenção. O fortalecimento da APS é essencial para atender às crescentes demandas de uma população com necessidades de saúde cada vez mais complexas.

Contudo, neste estudo a associação entre o uso do serviço de emergência e a multimorbidade não foi modificada pela avaliação do serviço como altamente orientado para a APS. Alguns fatores podem estar relacionados a este achado.

É fundamental considerar que as motivações para uso do serviço de emergência são complexas e multifacetadas, podendo ser influenciadas por características individuais e contextuais, conforme descrito na literatura^12–14^. Nesse contexto, a versão reduzida do PCATool-Brasil nos fornece informações acerca da orientação do serviço para a APS, mas não necessariamente sobre sua capacidade de resolução das necessidades de saúde do indivíduo. A adoção do ponto de corte do escore em 6,6, apesar de estabelecido pela literatura, também pode ter corroborado para esse achado, podendo não ser sensível o suficiente para detectar diferenças sutis em determinadas populações ou contextos.

A versão reduzida do PCATool-Brasil também apresenta limitações, tais como: a ausência de análise dos escores por atributo, o que dificulta a mensuração da presença e extensão dos atributos essenciais e derivados da APS, mesmo que por meio do escore geral; e a adaptação das perguntas do questionário, substituindo o termo "profissionais da APS" pelo termo "médico"^26,33^.

É importante ressaltar que, no instrumento da PNS 2019, todas as perguntas relacionadas ao PCATool-Brasil se referem ao médico que atendeu o indivíduo em sua última consulta na Unidade Básica de Saúde (UBS), e não necessariamente ao médico ou serviço ao qual ele é afiliado. Isso pode influenciar os resultados obtidos no sentido de suavização do efeito, especificamente se o último atendimento tiver ocorrido em outro local, onde este não possui afiliação.

Por fim, as perguntas foram aplicadas exclusivamente a pessoas que fizeram uso de UBS, ou seja, pessoas cobertas pela Saúde da Família. Nesse sentido, seria necessário que a qualidade tivesse um efeito muito superior para que fosse considerada como uma variável modificadora de efeito.

Entre as limitações do estudo, além das relacionadas à aplicação do PCATool-Brasil mencionadas anteriormente, destaca-se a utilização do desenho transversal, que impede a determinação de causalidade entre as variáveis estudadas. Adicionalmente, o período recordatório de duas semanas para o uso de serviços de emergência, podendo restringir a análise em longo prazo.

Apesar das limitações supracitadas, este estudo se distingue por explorar o efeito da cobertura e da orientação dos serviços para APS na relação entre multimorbidade e o uso de serviços de emergência, preenchendo uma lacuna importante na literatura, utilizando uma base de dados representativa da população brasileira.

O estudo evidencia que a cobertura de Saúde da Família é suficiente para modificar o efeito entre multimorbidade e utilização dos serviços de emergência, no sentido de reduzir o uso desses serviços por indivíduos com multimorbidade. No entanto, em um cenário complexo e multifatorial, a multimorbidade surge como um importante desafio para os sistemas de saúde, demandando um esforço coordenado para o seu enfrentamento. Assim, sugere-se a realização de novas pesquisas sobre esse tema, que contemplem a resolutividade do serviço de APS em si, visando compreender o seu impacto nos desfechos em saúde de indivíduos com multimorbidade.
